# Graphene nanoplatelets enhance neuronal differentiation of human bone marrow mesenchymal stem cells

**DOI:** 10.1186/s40659-025-00616-3

**Published:** 2025-05-30

**Authors:** Gulsah Sevimli, Eda Kus, Gulin Baran, Mahya Marashian, Nasrollah Tabatabaei, Nur Mustafaoglu

**Affiliations:** 1 Molecular Biology, Genetics and Bioengineering Program, Faculty of Engineering and Natural Sciences, Sabanci University, Istanbul, Turkey; 2 Department of Medical Nanotechnology, School of Advanced Technologies in Medicine, Tehran University of Medical Sciences, Tehran, Iran; 3 Sabanci University Nanotechnology Research and Application Center, Istanbul, Turkey

**Keywords:** Graphene nanoplatelet (GNP), Gelatin, Human bone marrow mesenchymal stem cells (hBMSCs), Neuronal differentiation

## Abstract

Stem cell technology plays a key role in advancing the understanding of neurological treatments and developing disease models that mimic human conditions. Differentiation of human bone marrow mesenchymal stem cells (hBMSCs) into neurons shows promise for treating neurodegenerative diseases. However, improving the functionality of these nerve cells remains a challenge. Graphene nanoplatelets (GNPs), with their excellent conductivity and biocompatibility, can enhance neuronal differentiation. This study examines the effect of GNPs on hBMSC differentiation. Cells cultured with varying GNP concentrations were assessed at 4 and 7 days using RT-qPCR and immunocytochemistry for neuronal markers MAP2, Nestin, and Tuj1. Results show that GNPs enhance marker expression and promote differentiation. Lower GNP concentrations maintained viability, while higher concentrations were detrimental. Morphological changes and increased fluorescence were observed with a 0.4 µg/ml GNP coating. Calcium imaging with Fluo4-AM indicated increased neuronal activity, underscoring GNPs’ role in neuronal maturation. These findings suggest GNPs can drive stem cell differentiation toward neurons, offering new therapeutic potential for neurodegenerative diseases.

**Graphical Abstract:**

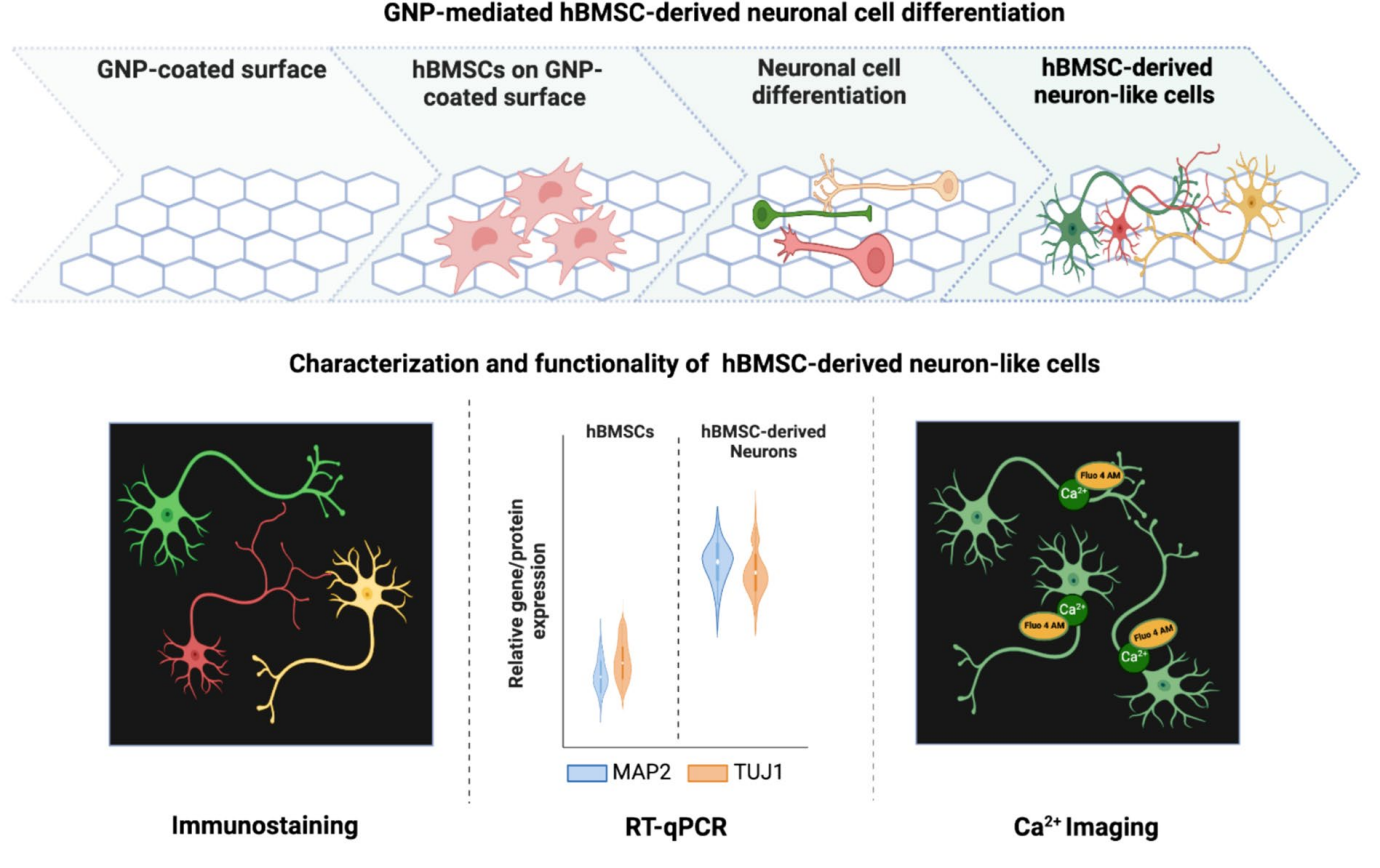

**Supplementary Information:**

The online version contains supplementary material available at 10.1186/s40659-025-00616-3.

## Introduction

Neurological diseases present complex pathologies that are challenging to accurately replicate in animal models, primarily due to inherent species differences and the limitations of these models in mimicking human disease processes [1, 2]. Alternatively, stem cell research has emerged as a pivotal field in tissue engineering, offering a promising avenue for disease modeling and the potential to repair or replace damaged tissues and organs [3, 4]. Among the various types of stem cells, human bone marrow mesenchymal stem cells (hBMSCs) hold significant promise due to their ability to differentiate into multiple cell types, including neurons [5, 6]. The neuronal differentiation of hBMSCs is critical for studying human-specific conditions, drug discovery/screening/development, personalized therapies, biomarkers, therapeutic targets, and pathways in neurological disorders [7–10]. However, several limitations exist in harnessing mesenchymal stem cells (MSCs) for neuronal differentiation and disease modeling, such as limited differentiation efficiency and maturation of neuronal phenotype [10]. Traditional differentiation methods, which often rely on chemical induction using growth factors, small molecules, and extracellular matrix components, are hindered by low efficiency, incomplete differentiation, and the need for prolonged culture periods [5, 6, 11, 12]. The search for innovative approaches that can enhance the differentiation process, while maintaining the viability and functional properties of the resulting neurons, is therefore critical for advancing stem cell-based technologies such as disease modeling and therapeutic approaches [13].

Graphene-based materials, including graphene oxide (GO) and reduced graphene oxide (rGO), have gained prominence in the fields of stem cell differentiation and tissue regeneration due to their remarkable mechanical strength, electrical conductivity, and surface area [14]. These materials are known to facilitate cell attachment, proliferation, and differentiation by interacting with cell surface receptors or through functionalization with bioactive molecules [15]. The significance of GO and rGO is underscored by their significant influence on neuronal differentiation and maturation, where they demonstrated high efficiency [16, 17]. Additionally, several studies have confirmed the versatility and efficacy of GO and rGO in scaffold development for neural tissue engineering and regenerative medicine [18]. These materials have shown promise in supporting the neuronal differentiation of stem cells and offer protection against enzymatic degradation during neural adhesion, differentiation, and growth [19–26]. Furthermore, composite materials that incorporate GO and rGO have been shown to subsequently enhance the functional properties required for neural stem cell differentiation, improve neuritogenesis, and facilitate nerve repair and regeneration [27–30]. Some research indicates that the incorporation of growth factors not only enhances the functional properties and bioactivity of GO but also improves the survival, proliferation, and differentiation of stem cells [31–34].Therefore, the integration of graphene-based materials represents a promising avenue for developing advanced neuronal models to generate new therapeutic strategies.

Graphene nanoplatelets (GNPs) exhibit distinct properties, such as superior electrical conductivity compared to GO and rGO, which make them particularly advantageous for neuronal cell culture [35]. Although there are a limited number of studies on the use of GNPs in biological applications, GNPs have notably been identified to be suitable scaffolds for promoting neuronal network development, as demonstrated in a study involving primary murine neurons [35]. Research has shown that composite microparticles combining chitosan and GNPs create robust three-dimensional scaffolds that significantly enhance mechanical strength, electrical conductivity, and stability for supporting the development of complex neuronal networks [36]. Similarly, cost-effective electroconductive scaffolds incorporating carboxy-functionalized GNPs into a polycaprolactone (PCL)-collagen matrix have been developed, which demonstrate a strong capability to promote the differentiation of mouse mesenchymal stem-like cells (m-MSC) into neurons under electrical stimulation [37]. Additionally, the development of a GelMA-based nano-bioink with GNPs has shown promise in supporting mouse neural stem cell survival, growth, and differentiation [38]. Furthermore, embedding GNPs into poly(3-hydroxybutyrate) scaffolds has proven effective in restoring normal electrophysiological function in primary neurons [35]. Beyond their structural advantages, GNPs are naturally biodegradable and biocompatible, making them suitable for integration with various scaffolds to enhance electrical stimulation. Their combination of electrical conductivity, mechanical strength, and thermal stability plays a crucial role in neurogenesis and neuritogenesis, particularly when applied in nerve tissue differentiation under electrical stimulation [39, 40]. Recent studies highlight that GNPs activate key signaling pathways, such as the MAPK/ERK pathway, during wireless electrical stimulation, leading to the upregulation of early growth response protein 1 (EGR1) [41]. Additionally, GNPs have been shown to influence crucial mechanisms involved in neuronal differentiation, including the axon guidance pathway, glutamatergic synapse pathway, calcium signaling pathway, and ECM-receptor interactions [41]. Collectively, these studies underscore the versatility and efficacy of GNP-reinforced biomaterials in advancing neural tissue engineering applications.

Despite the promising potential of GNPs, significant gaps remain in the current research on their use in stem cell differentiation. Much of the existing literature has primarily concentrated on the applications of GO and rGO, while the unique advantages of GNPs, particularly in the context of neuronal differentiation from human stem cells, have not been thoroughly explored. Here, this study is the first to address these gaps by systematically analyzing the concentration-dependent effects of GNPs on the differentiation of hBMSCs into neurons and to evaluate the combined effect of GNPs with a gelatin coating. The results of this study will provide valuable insights into the utility of GNPs as a novel strategy for enhancing stem cell-derived neuron generation, thereby advancing the field of neural tissue engineering.

## Materials and methods

### Materials

Human bone marrow mesenchymal stem cells (hBMSCs) were purchased from ATCC (PCS-500-012), and passages 7–15 were used during this study. Low glucose (1 g/l) Dulbecco’s Modified Eagle’s Medium (DMEM), penicillin/streptomycin, and l-glutamine were obtained from Gibco (New York, USA). Fetal bovine serum (FBS), obtained from Pan Biotech (Adienbach, Germany), was incorporated into the culture media at a concentration of 10% to support cell growth and viability. Trypsin and DPBS (w/and w/o Ca^2+^/Mg^2+^) were purchased from Pan Biotech (Adienbach, Germany). 3-(4,5-Dimethylthiazol-2-yl)−2,5-Diphenyltetrazolium bromide (MTT), 4′,6-diamidino-2-phenylindole (DAPI), and gelatin were obtained from Sigma Aldrich (Taufkirchen, Germany). Graphene nanoplatelets (GNPs) (xGnP^®^ Grade C) with a surface area of 750 m^2^/g were purchased from XG Sciences (Michigan, USA).

The neuron differentiation components, including IBMX (3-isobutyl-1-methylxanthine), B27 supplement, dibutyryl cyclic AMP (dcAMP), and hEGF, were sourced from Sigma Aldrich (Taufkirchen, Germany), while fibroblast growth factor-8 (FGF-8) was obtained from PeproTech (Waltham, USA). Nerve growth factor (NGF), recombinant human basic fibroblast growth factor (BFGF), and recombinant human brain-derived neurotrophic factor (BDNF) were purchased from R&D systems (Minnesota, USA).

Antibodies and conjugates, including Coralite488-conjugated goat anti-rabbit (#SA00013-2), Cy3-conjugated Affinipure goat anti-rabbit (#SA00009-1), MAP2 polyclonal antibody (#17490-1-AP), Nestin polyclonal antibody (#19483-1-AP), and TUBB3 monoclonal antibody (#66375-1-Ig), were sourced from Protein Tech (Chicago, USA).

### Cell culture

hBMSCs were cultured in a complete expansion medium containing low glucose DMEM supplemented with 2 mM l-glutamine, 10% FBS (heat-inactivated), and 1% penicillin/streptomycin in a humidified incubator at 37 °C with 5% CO_2_. After coating, 3 × 10^4^ cells/well were seeded into gelatin ± GNP coated wells of a 48-well plate in 200 µl of expansion media. At approximately 70–80% confluency, the cells were differentiated into neurons using neuron differentiation media. In parallel, 1 × 10^4^ cells/well were seeded and maintained with expansion media during the experiment as hBMSCs control.

### Gelatin and GNP coating

A 0.4% gelatin stock solution was prepared by dissolving gelatin in double-distilled water, followed by autoclaving the solution to ensure sterility. Before using, this stock solution was diluted to 0.2% in phosphate-buffered saline (PBS) to obtain the final working concentration for coating. Concurrently, a stock solution of GNPs (20 µg/ml) was prepared by dispersing the GNPs in dimethyl sulfoxide (DMSO) to ensure uniform distribution and prevent aggregation. GNPs were further diluted from this stock solution in PBS to achieve final concentrations of 0.1, 0.2, 0.4, 2, 5, and 10 µg/ml. Subsequently, to each of these GNP solutions, 0.2% gelatin (diluted from the 0.4% stock) was added and mixed thoroughly. Following the preparation of gelatin ± GNP solutions, 100 µl of each solution was added to individual wells of a 48-well plate before cell seeding. The plate was then incubated in a humidified incubator with 5% CO_2_ at 37 °C for 30 min. After incubation, any remaining solution was carefully aspirated from the wells, followed by washing with DPBS before cell seeding.

### Neuronal differentiation

**Fig. 1:**
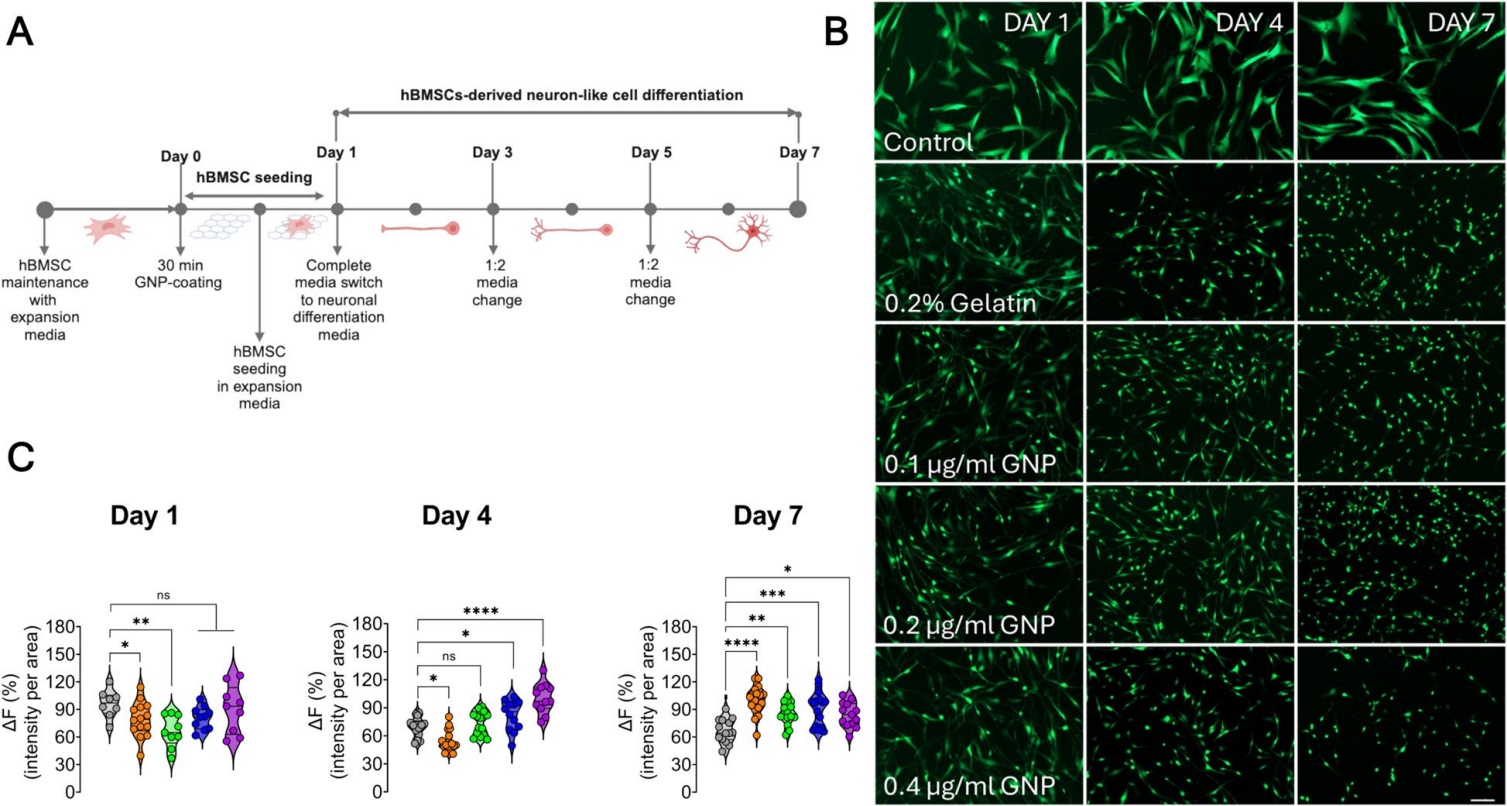
Tracking hBMSCs expressing GFP into neurons. **A** Differentiation protocol timeline. **B** Representative wide-field images of hBMSCs expressing GFP at day 1, 4, and 7 under different conditions: control, 0.2% gelatin, 0.1 µg/ml GNP, 0.2 µg/ml, and 0.4 µg/ml GNP. Scale bar: 100 µm (applies to all images). **C** Violin plots representing GFP fluorescence intensity over time. Sample sizes per condition: Day 1 control (*n* = 8/80), 0.2% gelatin (*n* = 14/139), 0.1 µg/ml (*n* = 9/90), 0.2 µg/ml (*n* = 10/100), and 0.4 µg/ml GNP (*n* = 9/90). Day 4 control (*n* = 14/122), 0.2% gelatin (18/181), 0.1 µg/ml GNP (*n* = 15/215), 0.2 µg/ml (*n* = 12/176), and 0.4 µg/ml GNP (*n* = 11/135). Day 7 control (*n* = 14/70), 0.2% gelatin (*n* = 18/90), 0.1 µg/ml GNP (*n* = 16/80), 0.2 µg/ml (*n* = 16/80), and 0.4 µg/ml GNP (*n* = 14/70). Dunnett’s test was applied to compare the gelatin ± GNP coating conditions relative to the control column. All values denote mean ± SEM. Asterisks indicate significance levels: * *p* < 0.1, ** *p* < 0.01, *** *p* < 0.001, **** *p* < 0.001

Neuron differentiation media was prepared according to the protocol outlined in the study [42] and followed the timeline in Fig. 1A. Briefly, 2 mM l-glutamine, 0.125 mM dcAMP, 0.5 mM IBMX, 20 ng/ml hEGF, 10 ng/ml (FGF-8), 10 ng/ml BDNF, 40 ng/ml NGF, 40 ng/ml BFGF, and 2% B27 were added to neurobasal media. The media was thoroughly mixed and filtered through a 0.22 µm syringe filter. At approximately 70–80% confluency after seeding, the neuron differentiation media was applied to a 7-day differentiation protocol, with half of the media replaced every 2 days.

### MTT assay

An MTT assay was prepared to evaluate the cytotoxicity of GNPs on hBMSCs. Initially, various concentrations of GNPs were prepared and coated onto the wells of a 96-well plate, as described in “Gelatin and GNP coating” section. Following this, hBMSCs were seeded into the coated wells at a density of 1 × 10^4^ cells/well. A control group, where cells were treated with a culture medium without GNPs, was also included. The cells were incubated for 1, 4, and 7 days at 37 °C with 5% CO_2_.

The MTT solution was prepared at a concentration of 5 mg/ml in DPBS and filtered through a 0.22 µm filter to remove any undissolved particles. After the treatment period, the medium was carefully removed from each well without disturbing the cells, and a fresh culture medium containing MTT solution (final concentration 0.5 mg/ml) was added to each well. The plate was then incubated for 3 h at 37 °C with 5% CO_2_ until purple formazan crystals were visible under a microscope.

Subsequently, the medium containing the MTT reagent was removed from each well, and 100 µl of DMSO was added and gently shaken for 10–15 min to dissolve the formazan crystals. Absorbance at 570 nm was measured using a microplate reader (Tecan Infinite M200 Pro). The average absorbance for each set of triplicate wells was calculated and then normalized to the absorbance of the control (no coating) to determine the percentage of cell viability for each GNP concentration relative to the control.

### Stable cell line generation

The HEK293T cell line was utilized to produce lentivirus using the LeGO-G2-PURO plasmid construct to generate a stable cell line (Supplementary Figure 3A). This construct encodes both a green fluorescence protein (GFP) reporter gene and a puromycin resistance gene for selection.

Twenty-four hours before transfection, HEK293T cells were seeded in a 6-well plate at a density of 6 × 10^4^ cells/well in complete media, consisting of high glucose DMEM supplemented with 10% FBS, 1% l-glutamine, and 1% penicillin–streptomycin. The following day, the cells were starved in complete DMEM containing 5% FBS for 4 h.

For the transfection process, PEI-MAX (1 µg/ml) transfection reagent was used with a total of 3 µg plasmid DNA per well, at a 1:4 DNA to PEI-MAX ratio. To produce the virus, 1.5 µg of LeGO-G2-PURO was mixed with packaging plasmids—0.75 µg pMDLg/pRRE, 0.45 µg pRSV-rev, and 0.3 µg pCMV-VSVg—in 250 µl of serum-free DMEM, and the mixture was gently vortexed. This plasmid-DNA mixture was then combined with 12 µg of PEI-MAX using vortexing and incubated for 30 min at room temperature. After incubation, the mixture was added drop by drop to the HEK293T cells, and the cells were incubated with the transfection mixture for 16 h.

The following day, the media was replaced with fresh complete DMEM containing 10% FBS. The media was changed daily for 2 days, and the lentivirus-containing media was collected 24- and 48 h post-transfection. The collected virus was filtered through a 0.44 µm filter and stored at 4 °C for short-term storage or at −20 °C for long-term storage.

hBMSCs were seeded 1 day before infection in a 6-well plate with 2.5 × 10^5^ cells/well. On the following day, the cells were incubated with the filtered LeGO-G2-PURO virus for 16 h. After incubation, the infected and GFP-expressing hBMSCs were selected in 0.5 µg/ml puromycin within standard hBMSCs expansion media. Following a selection period of 10–21 days, the cells were used for the tracking experiment. Stable cell lines were imaged using an inverted wide-field epifluorescence microscope (Zeiss Axio Observer Z1/7, Carl Zeiss AG, Oberkochen, Germany), equipped with an LED light source (Colibri 7) and an LD A Plan 10×/0.25 Ph 1 dry objective. Fluorescence signals were captured by exciting cells at 488 nm with emission filters set to 500–550 nm.

### Flow cytometry

Flow cytometry was used to analyze GFP expression in hBMSCs from passages 7, 11, and 15. Cells were harvested by trypsinization (0.05% trypsin–EDTA), neutralized with media, and centrifuged at 300×*g* for 5 min before resuspending in PBS. Cell suspensions were adjusted to 2 × 10^5^ cells/ml to ensure a single-cell analysis. 500 µl of each sample was transferred to flow cytometry tubes and analyzed using a CytoFLEX flow cytometer (Beckman Coulter, USA) equipped with a 488 nm laser. GFP fluorescence was detected using a FITC filter. The gating strategy included an FSC vs. SSC plot to exclude debris and a FITC histogram to analyze GFP expression. 10,000 events were recorded per sample, with PMT voltages optimized to place GFP-negative control fluorescence in the first decade of the histogram. GFP expression was quantified as the percentage of GFP-positive cells.

### Reverse transcription quantitative polymerase chain reaction (RT-qPCR)

Total RNA was extracted using TRIzol reagent (Thermo Fisher, 1596026) according to the manufacturer’s instructions. cDNA was reverse transcribed from total RNA using oligo (dT) primers (ABM, G236). SYBR Green Master Mix (ABM, G891) and LightCycler 480 (Roche) were used for two-step RT-qPCR reactions with the following cycle conditions: 40 cycles of 95 °C for 15 s and 60 °C for 1 min. Changes in mRNA levels were quantified using the 2^−ΔΔCT^ method, with GAPDH mRNA as the control. The primers used during the study were as follows: MAP2 5′CTCAGCACCGCTAACAGAGG3′; and 5′CATTGGCGCTTCGGACAAG3′; Tuj1 5′GGCCAAGGGTCACTACACG3′; and 5′GCAGTCGCAGTTTTCACACTC3′; Nestin 5′CTGCTACCCTTGAGACACCTG3′; and 5′GGGCTCTGATCTCTGCATCTAC3′; GAPDH 5′CACCGTCAAGGCTGAGAACG3′; and 5′GCCCCACTTGATTTTGGAGG3′. Reactions were performed in duplicates.

### Cell immunofluorescence

The cell culture media was removed after coating and seeding the cells according to “Cell culture” and “Gelatin and GNP coating” sections. On the 4 th and 7 th days of differentiation, the cells were fixed using 200 µl of cold methanol (MeOH) for 20–30 min at room temperature in the dark. Following fixation, cells were washed three times with DPBS (+/+). To block non-specific binding sites, cells were incubated with a blocking buffer (1.5% Bovine Serum Albumin + 0.01% Tween80 in PBS) for 1 h at room temperature. Primary antibodies MAP2 (#17490-1-AP), Tuj1 (#66375-1-Ig), and Nestin (#19483-1-AP) were diluted in the blocking buffer to final concentrations of 1:500, 1:200, and 1:200, respectively. Cells were incubated with these primary antibodies overnight at 4 °C. After the overnight incubation, the cells were washed three times with DPBS (+/+) to remove any unbound primary antibodies. Cy3-conjugated and FITC-conjugated secondary antibodies were diluted in the blocking buffer. Cells were incubated with the secondary antibody solution for 1 h at room temperature in the dark. After incubation with the secondary antibodies, the cells were washed three times with DPBS (+/+). The cells were incubated with DAPI for 10 min at room temperature in the dark to stain the nuclei. Immunostained cells were imaged using the same epifluorescence microscope equipped with an LD A Plan 20×/0.35 Ph 1 dry objective. Fluorescence signals were captured by exciting cells at 548 nm (Cy3), 488 nm (FITC), and 353 nm (DAPI), with emission filters set to 560–590 nm, 500–550 nm, and 420–470 nm, respectively. To minimize photobleaching, exposure time and light intensity were carefully controlled.

### Calcium imaging

Fluo-4 AM was dissolved in DMSO to create a 1 mM stock solution. A working solution (5 µM) was prepared in HBSS (w/o Ca^2+^ and Mg^2+^). After coating and seeding the cells, the cell culture media was removed, and the cells were washed twice with HBSS (w/o Ca^2+^ and Mg^2+^). Fluo-4 AM working solution was added to the cells, which were then incubated at 37 °C for 30 min in the dark. After incubation, the Fluo-4 AM solution was removed, and the cells were washed three times with HBSS (with Ca^2+^ and Mg^2+^) to remove any excess dye. The cells were equilibrated for 30 min at room temperature in the dark to ensure complete de-esterification of the dye. The cells were then placed on the stage of a fluorescence microscope. The cells were excited at 494 nm and emission was collected at 516 nm. Photobleaching was minimized by limiting exposure and intensity. Images were acquired using the same epifluorescence microscope, equipped with an LD A-Plan 10×/0.25 Ph 1 dry objective. Fluorescence signals were captured by exciting cells at 488 nm, with emission detected at 509 nm.

### Statistical analysis

Image analysis was conducted using GraphPad Prism software version 9.1.2 (GraphPad Software, San Diego, CA, USA). All experiments were conducted in triplicate, with experimental repeats denoted as ‘*N*’, and the number of analyzed cells per conditions as ‘*n*’. For example, 3/11 indicates *N* = 3 (triplicate cultures) and *n* = 11 (individual cells analyzed per experiment). ΔF_intensity_ was calculated as (1 − (F/F_0_) * 100), where F_0_ represents baseline fluorescence intensity. For relative comparison across multiple groups, the maximum signal was set to 100%, and all other groups were normalized, accordingly. Statistical comparison of two groups was evaluated using a two-tailed Student *t* test. Statistical comparisons of multiple groups were performed using one-way ANOVA, followed by either Dunnett’s post-test (comparison to control) or Bonferroni’s post-test (comparison between all groups).

## Results

### Cytotoxicity of GNP on hBMSCs

To evaluate the cytotoxicity of graphene nanoplatelets (GNPs) on hBMSCs, we conducted MTT assays at three different time points—day 1, day 4, and day 7—of cell culture. On day 1, cell viability was relatively high for treatments with 0.2% gelatin and GNP concentrations from 0.1 to 0.4 µg/ml compared to the control group of no GNP treatment (Supplementary Figure 1A). However, higher GNP concentrations resulted in a notable decline in cell viability (Supplementary Figure 1A). By day 4, cell viability improved in both the control and 0.2% gelatin groups (Supplementary Figure 1B). Additionally, cells exposed to 0.1 µg/ml and 0.2 µg/ml GNP showed increased viability compared to day 1, although higher GNP concentrations continued to reduce cell viability (Supplementary Figure 1B). On day 7, high GNP concentrations sustained a decrease in cell viability, while the control and 0.2% gelatin groups exhibited reduced viability (Supplementary Figure 1C). Conversely, cells coated with 0.1 µg/ml and 0.2 µg/ml GNPs maintained high cell viability (Supplementary Figure 1C). Additionally, MTT assay results displayed that DMSO used to homogenize GNP solutions affected hBMSCs viability in a concentration-dependent manner (Supplementary Figure 2A–C). DMSO concentrations in GNP solutions of 0.1 µg/ml, 0.2 µg/ml, and 0.4 µg/ml did not significantly alter cell viability at any time point, but higher DMSO concentrations resulted in a meaningful decrease in cell viability (Supplementary Figure 2A–C). All these results suggest that while lower concentrations of GNP and DMSO may not significantly impact cell viability, higher concentrations can have detrimental effects, particularly over extended periods. Based on the cytotoxicity results, we selected concentrations of 0.1 µg/ml, 0.2 µg/ml, and 0.4 µg/ml GNP for further experiments, as they demonstrated the most favorable balance between minimal cytotoxicity and effective differentiation induction of hBMSCs.

### Tracking of differentiation of hBMSCs into neurons

We next tracked the differentiation of hBMSCs into neurons by monitoring GFP expression using GFP-expressing hBMSCs which were created by infecting hBMSCs with LeGO-G2-PURO virus. The main purpose of using this construct (Supplementary Figure 3A) and lentiviral infection in this study is for tracking the GFP to follow the differentiation process from hBMSCs to neuron by creating stable GFP expression via lentiviral infection on hBMSCs. LeGO-G2-PURO virus does not have any other gene to manipulate differentiation processes except insertion of EGFP gene to observe differentiation, to follow morphological changes, and to evaluate fluorescence intensity change per area over the duration. To confirm successful lentiviral transduction of hBMSCs with the Lego-G2-PURO construct, flow cytometry was performed to assess GFP expression levels. The result indicates a high percentage of GFP-positive cells (about 85–90%), confirming efficient viral transduction and stable expression (Supplementary Figure 3B). GFP expression was analyzed in hBMSCs across multiple passages to ensure stable fluorescence retention over time. The results show consistent GFP expression, indicating that the lentiviral transduction was stable and did not diminish over subsequent passages (Supplementary Figure 3B). Bright-field (BF), GFP, and merged images with different magnifications (20× and 63×) further verified the presence of GFP-expressing hBMSCs, showing clear and uniform fluorescence distribution within the cell cytoplasm (Supplementary Figure 3C, D). In addition, cell morphology remains unchanged, confirming that lentiviral transduction does not alter the typical spindle-like hBMSC shape.

With GFP-expressing hBMSCs, hBMSC-derived neuron differentiation was tracked under various coating conditions (0.2% gelatin, 0.1 µg/ml, 0.2 µg/ml, and 0.4 µg/ml of GNP in 0.2% gelatin) over 7 days and compared with the hBMSCs without any treatment of differentiation media (control group). Figure 1B illustrates the progression and morphological changes of hBMSCs into neurons with different coatings. Here, the most important morphological change observed when stable hBMSCs turn into neurons is the elongation of the cells. From day 1 to day 7, the large flattened or spindle-like shapes of hBMSCs under all coatings became smaller, more elongated, and developed extended projections, resembling neuron-like structures (Fig. 1B; Supplementary Figure 4). Control cells retained their spindle-like morphology with minimal elongation, while 0.2% gelatin and 0.4 µg/ml GNP-coated cells exhibited the most pronounced neurite extension, indicating enhanced differentiation in these conditions. 0.1 and 0.2 µg/ml GNP led to moderate elongation, suggesting a dose-dependent response to GNP coatings over time (Fig. 1B; Supplementary Figure 4). We also quantified the percentage of fluorescence intensity per area (Fig. 1C). On day 1, the violin plot shows that 0.4 µg/ml GNP coating significantly increased the fluorescence intensity per area compared to the control and 0.2% gelatin coating, while 0.1 µg/ml and 0.2 µg/ml NP coatings showed decreased intensity (Fig. 1C). By day 4, 0.1 µg/ml and 0.2 µg/ml GNP coatings increased fluorescence intensity compared to the control and 0.2% gelatin, but this increase was less pronounced than with 0.4 µg/ml GNP coating. On day 7, the presence of 0.2% gelatin alone or in combination with GNP significantly enhanced fluorescence intensity compared to the control. We observed the highest fluorescence intensity with the 0.2% gelatin coating alone. However, the addition of GNP at 0.1 µg/ml, 0.2 µg/ml, and 0.4 µg/ml also resulted in significantly higher fluorescence intensities compared to the control, though slightly lower than the 0.2% gelatin coating alone. These results indicate that both 0.2% gelatin and GNP coatings, especially at 0.4 µg/ml, promote the differentiation of hBMSCs into neuron-like cells, as evidenced by increased fluorescence intensity and morphological changes over 7 days.

### RT-qPCR and immunostaining analysis of neuronal biomarkers in hBMSCs

**Fig. 2:**
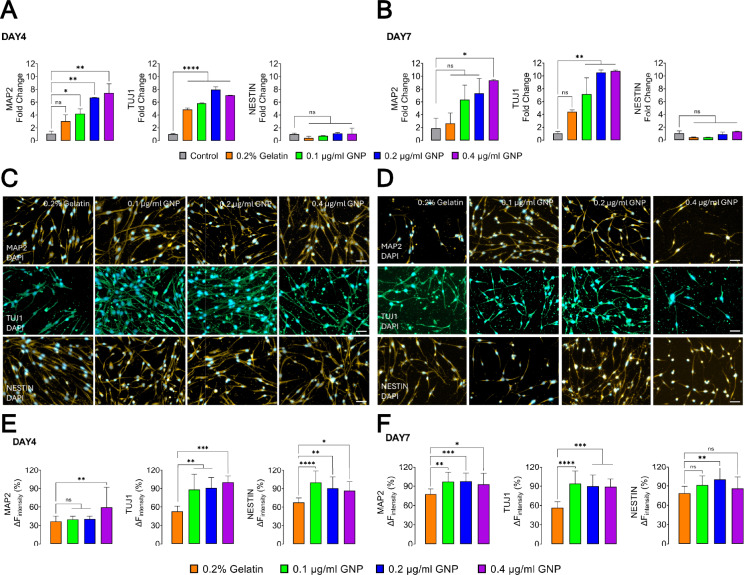
Enhanced neuronal marker expression in hBMSCs cultured on GNP-coated surfaces. **A**, **B** Quantitative RT-PCR fold change results for neuronal markers (MAP2, Tuj1, and Nestin) on days 4 and 7. **C**, **D** Representative immunofluorescence images of DAPI co-staining with MAP2, Tuj1, and Nestin on days 4 and 7, respectively. Scale bars: 50 µm (applies to all images). **E**, **F** Quantification of immunostaining results, indicating the percentage of marker-positive cells on days 4 and 7. Sample sizes per condition: Day 4 (**E**) MAP2: 0.2% gelatin (*n* = 11/113), 0.1 µg/ml (*n* = 13/146), 0.2 µg/ml (*n* = 11/133), and 0.4 µg/ml (*n* = 10/145). Tuj1: 0.2% gelatin (*n* = 9/46), 0.1 µg/ml (*n* = 10/79), 0.2 µg/ml (*n* = 10/56), and 0.4 µg/ml (*n* = 10/88). Nestin: 0.2% gelatin (*n* = 10/101), 0.1 µg/ml (*n* = 11/112), 0.2 µg/ml (*n* = 11/112), and 0.4 µg/ml (*n* = 10/118). Day 7 **F** MAP2: 0.2% gelatin (*n* = 11/149), 0.1 µg/ml (*n* = 17/186), 0.2 µg/ml (*n* = 9/123), and 0.4 µg/ml (*n* = 10/145). Tuj1: 0.2% gelatin (*n* = 6/54), 0.1 µg/ml (*n* = 6/54), 0.2 µg/ml (*n* = 6/48), and 0.4 µg/ml (*n* = 5/29). Nestin: 0.2% gelatin (*n* = 12/146), 0.1 µg/ml (*n* = 14/202), 0.2 µg/ml (*n* = 11/155), and 0.4 µg/ml (*n* = 9/148). Dunnett’s test compared the GNP-coated conditions relative to the 0.2% gelatin. All values represent mean ± SEM. Asterisks indicate significance levels: * *p* < 0.1, ** *p* < 0.01, *** *p* < 0.001, **** *p* < 0.001

Our results so far demonstrate that GNP coating is essential for effective morphological differentiation of hBMSCs. However, the spatial distribution and quantification of biomarkers at both mRNA and protein levels require further investigation. To address this, we initially performed an RT-qPCR for key neuronal differentiation markers (MAP2, Nestin, and Tuj1) in hBMSCs under various coatings. On day 4, GNP coatings increased MAP2 expression, a marker of mature neurons, in a dose-dependent manner, indicating enhanced neuronal differentiation (Fig. 2A). Tuj1 expression showed a highly significant increase in all GNP-treated conditions, with the highest expression at 0.2 µg/ml GNP. In contrast, Nestin expression, a neural progenitor marker, remained similar across all groups, with no significant differences observed (Fig. 2A). By day 7, MAP2 expression remained significantly higher in the 0.4 µg/ml GNP group, suggesting sustained neuronal differentiation (Fig. 2B). Tuj1 expression continued to show a strong dose-dependent increase, with significantly higher levels at all GNP included groups compared to the control (no differentiation) and 0.2% gelatin. In contrast, Nestin expression remained low across all conditions, with no significant differences between groups (Fig. 2B).

Next, we used a high-resolution immunofluorescence assay to visually confirm the protein expression and localization of these biomarkers within the cells, exploring their full potential and underlying mechanisms. Immunofluorescence analysis at day 4 revealed that GNP coatings enhance early neuronal differentiation in hBSMCs. MAP2 expression significantly high in 0.4 µg/ml GNP-treated groups at day 4, and the MAP2 expression significancy was observed at all GNP-treated conditions at day 7, as observed in immunofluorescence staining and confirmed by quantification (Fig. 2C, E). This may indicate the neuronal maturation over time. The 0.2% gelatin exhibited weaker MAP2 expression, suggesting minimal neuronal commitment. Similarly, Tuj1 expression showed a significant increase across all GNP-treated conditions, further supporting continuation of neuronal differentiation. Additionally, cells in GNP-treated groups displayed elongated neurite-like projections, particularly in the 0.4 µg/ml GNP condition, suggesting early neurite outgrowth (Fig. 2C). Nestin expression remained relatively similar across all groups in immunofluorescence staining but was significantly upregulated in GNP-treated conditions (Fig. 2E). This suggests that at day 4, hBMSCs still retain neural progenitor characteristics, even in the presence of GNPs. GNP coatings continued to promote neuronal differentiation and maturation on day 7 in hBMSCs (Fig. 2D, F). MAP2 expression remained significantly higher in all GNP-treated groups compared to 0.2% gelatin, suggesting sustained neuronal differentiation (Fig. 2F). Tuj1 expression was also significantly high in all GNP groups, indicating enhanced neuronal network formation. Notably, Nestin expression decreased across all groups, yet quantification showed a slight but significant difference in 0.2 µg/ml GNP-treated condition (Fig. 2F). This suggests that while some cells may still retain progenitor characteristics, the overall trend favors neuronal maturation rather than progenitor maintenance. These findings confirm that GNP coatings facilitate neuronal differentiation in a dose- and time-dependent manner, with increased MAP2 and Tuj1 expressions supporting neuronal maturation and network development, while decreasing Nestin levels indicate a shift away from the progenitor state. Among all conditions, 0.4 µg/ml GNP remains the most effective in promoting neuronal differentiation at this later stage.

We showed in Fig. 2C and 2D representative immunostaining images of hBMSCs on days 4 and 7 of differentiation, highlighting the expression of neuronal markers MAP2, Tuj1, and Nestin. The intensity of all neuronal biomarkers robustly increased over time by indicating a clear progression and neuronal differentiation (Fig. 2C, D). 0.4 µg/ml GNP coating exhibited the highest MAP2 expression at day 4 (Fig. 2E). Lower concentrations (0.1 and 0.2 µg/ml GNP) also increased MAP2 expression compared to 0.2% gelatin alone, but to a lesser extent than 0.4 µg/ml GNP (Fig. 2E). The significant expression of MAP2 observed across all GNP conditions (Fig. 2F) may indicate a dose-dependent effect of GNPs on neuronal maturation over time. Tuj1 expression increased with GNP addition, while 0.1 µg/ml GNP showed the highest Nestin expression, indicating a greater presence of progenitor cells at this concentration (Fig. 2E). GNP coatings continued to promote neuronal differentiation and maturation in hBMSCs on day 7 (Fig. 2D, F). MAP2 expression remained significantly higher in the 0.2 and 0.4 µg/ml GNP-treated groups compared to the 0.2% gelatin control, suggesting sustained neuronal differentiation (Fig. 2F). Similarly, Tuj1 expression significantly increased in the 0.2 and 0.4 µg/ml GNP groups, indicating enhanced neuronal network formation. Notably, Nestin expression did not differ significantly from the control condition; however, quantification revealed a slight but statistically significant increase in the 0.2 µg/ml GNP-treated group (Fig. 2F). This observation suggests that while some cells may retain progenitor characteristics, the overall trend favors neuronal maturation rather than progenitor maintenance. Collectively, these findings demonstrate that GNP coatings significantly enhance the differentiation of hBMSCs into neurons, as evidenced by increased MAP2 and Tuj1 expression, supporting both neuronal maturation and network development.

### Ca^2+^ imaging of differentiated neurons

**Fig. 3:**
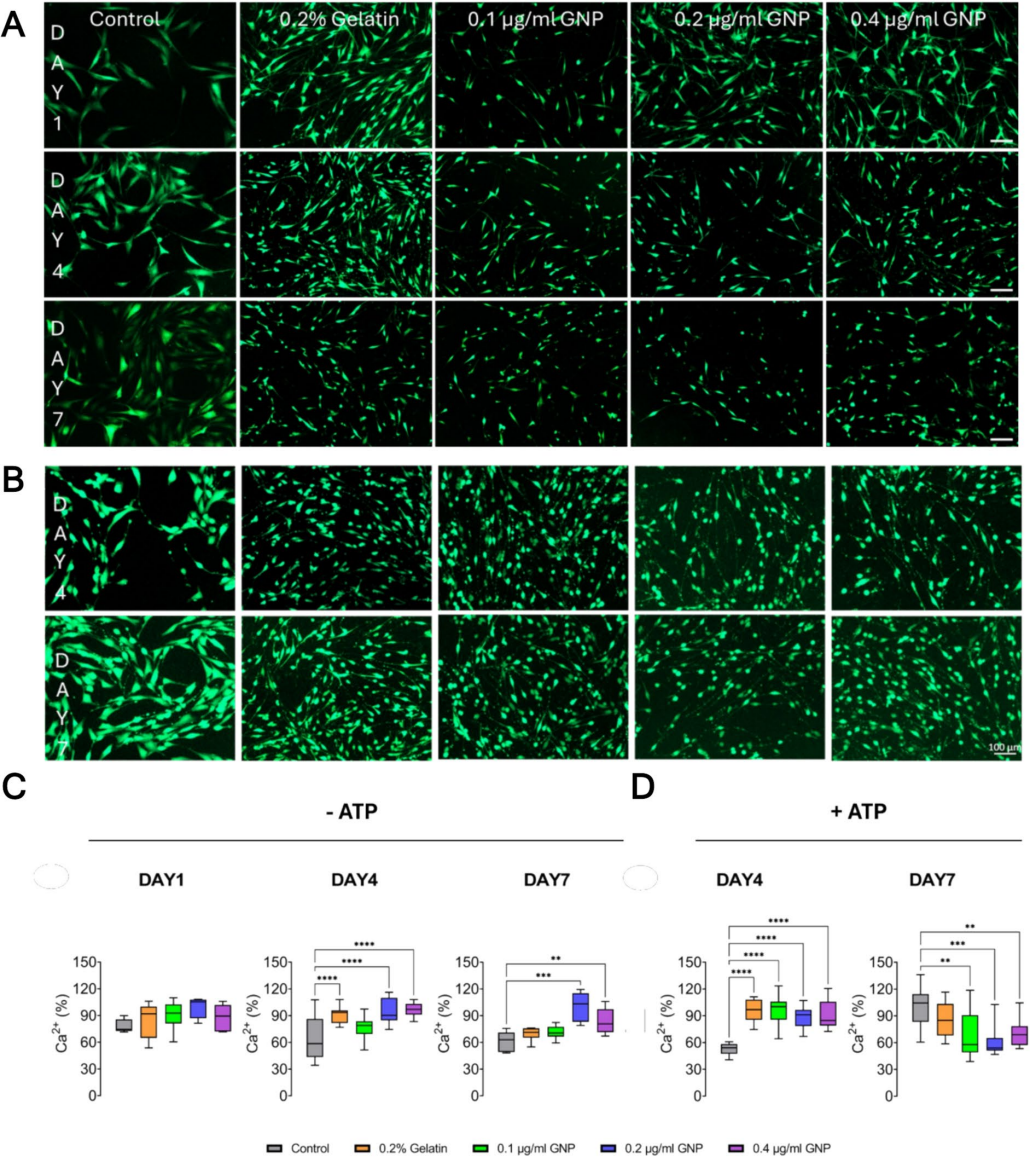
Ca^2+^ fluctuations in differentiated hBMSCs on GNP-coated surfaces using Fluo-4 AM. Representative wide-field images of hBMSC-derived neurons at day 1, 4, and 7. **A** Without ATP stimulation and **B** with ATP stimulation (100 100 µM, 5 min) under different conditions. Scale bars: 100 µm (applies to all images). **C** Quantification of calcium intensity at days 1, 4, and 7 without any ATP stimulation. Sample sizes per condition: Day 1 control (*n* = 5/50), 0.2% gelatin (*n* = 6/60), 0.1 µg/ml GNP (*n* = 7/70), 0.2 µg/ml GNP (*n* = 4/40), and 0.4 µg/ml GNP (*n* = 6/60). Day 4 control (*n* = 13/65), 0.2% gelatin (*n* = 13/130), 0.1 µg/ml GNP (*n* = 17/167), 0.2 µg/ml GNP (*n* = 17/170), and 0.4 µg/ml GNP (*n* = 15/150). Day 7 control (*n* = 5/50), 0.2% gelatin (*n* = 6/60), 0.1 µg/ml GNP (*n* = 6/60), 0.2 µg/ml GNP (*n* = 5/50), and 0.4 µg/ml GNP (*n* = 7/70). **D** Intracellular Ca.^2+^ intensity following ATP stimulation in differentiated hBMSCs. Sample sizes per condition: Day 4 control (*n* = 10/100), 0.2% gelatin (*n* = 13/130), 0.1 µg/ml GNP (*n* = 12/120), 0.2 µg/ml GNP (*n* = 12/120), and 0.4 µg/ml GNP (*n* = 15/150). Day 7 control (*n* = 10/100), 0.2% gelatin (*n* = 10/100), 0.1 µg/ml GNP (*n* = 13/130), 0.2 µg/ml GNP (*n* = 12/120), and 0.4 µg/ml GNP (*n* = 10/100). Dunnett’s test was applied to compare the gelatin ± GNP coating conditions relative to the control. Data are presented as mean ± SEM. Asterisks indicate significance levels: * *p* < 0.1, ** *p* < 0.01, *** *p* < 0.001, **** *p* < 0.001

We next sought to evaluate the calcium responses in hBMSC-derived neurons to assess their functionality using Fluo4-AM before and after ATP stimulation over time under different GNP-coated conditions. Figure 3A shows positively stained cells with Fluo4-AM under different coating conditions including 0.2% gelatin, 0.1, 0.2, and 0.4 µg/ml GNP in 0.2% gelatin. On day 1, cells exhibited a spindle-like morphology across all conditions, with no clear neuronal features or structural specializations. By day 4, GNP-treated groups displayed elongated neurites with an increasingly branched and interconnected network, whereas the control and gelatin groups retained a more spread-out, less connected morphology. Following ATP stimulation, noticeable accumulations of Ca^2+^ signals along neurites were observed in the 0.2 and 0.4 µg/ml GNP conditions, particularly in axon-like projections, suggesting functional neuronal activation (Fig. 3B). These accumulations indicate potential localized Ca^2+^ signaling in axonal compartments, a feature commonly associated with synaptic activity and neuronal excitability. In contrast, the control and gelatin groups showed diffuse fluorescence distribution, with less defined accumulations, suggesting reduced or impaired Ca^2+^ signaling responses.

We observed a strong correlation between GNP concentration and neuronal activity. On day 1, Ca^2+^ intensity remained low across all conditions, with no significant differences (Fig. 3C). By day 4, a significant increase was observed not only in the 0.2 and 0.4 µg/ml GNP groups but also in the 0.2% gelatin group (Fig. 3C). This suggests that while gelatin alone may provide a moderate increase in Ca^2+^ signaling. (Fig. 3C). On day 7, Ca^2+^ intensity remained significantly higher in the 0.2 and 0.4 µg/ml GNP groups, while the gelatin group did not show a sustained increase, indicating that GNP coatings play a more prominent role in long-term neuronal activity (Fig. 3C). On day 4, ATP stimulation induced a significant increase in Ca^2+^ response across multiple conditions (Fig. 3D). Notably, the 0.2% gelatin group exhibited a strong ATP-induced Ca^2+^ transient, comparable to the responses observed in the 0.2 and 0.4 µg/ml GNP groups (Fig. 3D). This suggests that gelatin alone can enhance ATP-triggered Ca^2+^ signaling responses. By day 7, the control and 0.2% gelatin groups displayed significantly increased ATP-induced Ca^2+^ transients, while 0.2 and 0.4 µg/ml GNP groups exhibited a reduced response compared to day 4, indicating potential adaptation or homeostatic regulation of calcium influx over time (Fig. 3D). These findings indicate that gelatin alone can temporarily enhance ATP-induced Ca^2+^ signaling at early stages, but GNP coatings provide a more stable and sustained enhancement of neuronal excitability over time. The attenuated response in the 0.4 µg/ml GNP group at day 7 may reflect a regulation of intracellular calcium homeostasis as neurons mature, which could be a sign of advanced functional adaptation.

## Discussion

In this study, we investigated GNP’s effect on the differentiation of hBMSCs into neurons. Our experiments unveiled that potent GNP coating in effective concentration contributed to human MSC differentiation into neurons effectively. Gelatin, a collagen-derived biopolymer, is widely used in tissue engineering due to its excellent biocompatibility, biodegradability, and cell adhesion properties [43–45]. The presence of RGD sequences in gelatin facilitates integrin-mediated cell adhesion, which is essential for stem cell proliferation and differentiation [46, 47]. In our study, gelatin was used in conjunction with GNPs to create a synergistic environment for the neuronal differentiation of human bone marrow mesenchymal stem cells (hBMSCs). While GNPs provide mechanical strength and electrical conductivity, gelatin offers a biocompatible and bioactive surface that supports cell adhesion and growth. In the first step, we observed that the percentage of cell viability of hBMSCs decreased as the concentration of GNP increased. The observed cytotoxicity patterns align with the literature, which indicates that the impact of GNPs on cell viability is highly concentration-dependent [48]. Lower concentrations of GNPs are generally tolerated by cells, but higher concentrations tend to induce cytotoxic effects, as seen in this study. The initial high cell viability at day 1 suggests that hBMSCs can endure GNP exposure in the short term, especially when combined with gelatin, which likely provides a protective and supportive environment. However, the continued exposure, particularly at higher GNP concentrations, leads to a decline in cell viability, consistent with reports that prolonged exposure to higher levels of graphene-based materials can compromise cell health [37]. The improvement in cell viability by day 4 in the control and low GNP concentration groups suggests that cells may partially adapt to the GNPs or recover from initial stress. Conversely, the persistent reduction in viability at higher GNP concentrations highlights their potential cytotoxicity over time, as excessive GNP exposure might disrupt cellular functions and induce oxidative stress.

Building on the established understanding of GNPs’ concentration-dependent effects on cell viability, we next examined their influence on the differentiation of hBMSCs into neurons under 0.1, 0.2, and 0.4 µg/ml GNP with 0.2% gelatin coatings. To ensure long-term tracking of hBMSCs during neuronal differentiation, we employed a lentiviral transduction strategy using a LeGO-G2-PURO vector, which expresses EGFP (enhanced green fluorescent protein) under the SFFV promoter. Flow cytometry analysis and microscopy images confirmed that a high percentage of cells successfully expressed GFP. The retention of GFP positivity across passages confirms successful genomic integration and transcriptional stability in hBMSCs. Microscopy images show that GFP-expressing hBMSCs retain their typical spindle-like morphology, indicating that lentiviral transduction does not interfere with normal cell function. The high efficiency of transduction and low variability in GFP expression demonstrate the reliability of this method for long-term cell tracking. The results suggested that the differentiation of hBMSCs into neurons can be significantly influenced by the extracellular environment, including the use of specific coatings like gelatin and graphene nanoplatelets (GNPs), as hBMSCs undergo morphological changes due to differentiation in the presence of GNPs. While GFP expression remained stable across passages, fluorescence intensity per area varied significantly depending on differentiation in the presence of GNP treatment. Cells are more spread out at day 1, meaning the fluorescence intensity is distributed over a larger area. But cells become smaller and more elongated at day 4 and 7. In addition to this, higher cell density leads to increased fluorescence intensity. GNPs can also support cell attachment and proliferation, leading to denser cultures and enhanced fluorescence detection [49].

Expanding on the recognized influence of GNPs on cell viability and morphological differentiation, we proceeded to examine how GNP coatings affect the molecular markers associated with neuronal differentiation in hBMSCs. GNPs have been shown to enhance neuronal differentiation by modulating the expression of key neuronal markers. The increase in MAP2 and Tuj1 expression across various GNP concentrations supports that GNPs can promote neuronal maturation and differentiation. The dose-dependent increase in MAP2 expression suggests that higher GNP concentrations more effectively drive MSCs toward a neuronal phenotype, which is in line with studies highlighting the role of GNPs in enhancing neurogenic differentiation [37]. MAP2 was significantly upregulated in 0.4 µg/ml GNP-coated groups, reinforcing its role in promoting neuronal commitment. Tuj1 expression exhibited a strong dose-dependent increase across all GNP-treated groups, with the highest expression at 0.4 µg/ml GNP, suggesting that GNPs enhance early neuronal differentiation and network formation. This is consistent with studies demonstrating the role of graphene derivatives in enhancing neurogenic differentiation. For instance, scaffolds containing graphene-based nanomaterials have been shown to induce neuronal differentiation due to their unique structural features like porosity and wrinkles [50]. The reduction in Nestin expression, particularly at higher GNP concentrations and over time, supports the notion that GNPs facilitate the transition from progenitor to mature neuron-like cells. While Tuj1 and MAP2 serve as early and mature neuronal markers, respectively, Nestin remains a neural progenitor marker, meaning that a fully differentiated neuronal population should exhibit minimal Nestin expression. Indeed, in our study, some Nestin expression persisted in cells adjacent to hBMSCs, despite the increased expression of Tuj1 and MAP2 in neuron-like cells under 0.2% gelatin and varying GNP concentrations, suggesting that the differentiated population is heterogeneous, including early and mature neuron populations. This is consistent with previous studies demonstrating that iPSC-derived and ESC-derived neuronal differentiation results in a mixed population of mature, immature, and metabolically abnormal neuronal cells rather than a completely synchronized in vivo-like developmental process [51, 52]. Similarly, our hBMSC-derived neurons exhibit a range of differentiation states, further highlighting that stem cell-based differentiation systems inherently generate heterogeneous neuronal populations. Notably, this heterogeneity may serve as an advantage for modeling neurological diseases, as neurologically diseased brains consist of a mix of mature, immature, and neuroprogenitor cells due to impaired neurogenesis [53]. Thus, while our differentiation model does not perfectly mimic in vivo development, it provides a physiologically relevant system for in vitro applications, particularly in disease modeling.

Following the established insights into GNPs’ concentration-dependent effects on cell viability, their ability to drive morphological differentiation, and their influence on molecular markers of neuronal differentiation, our next focus was to assess the functional maturation of hBMSC-derived neurons by monitoring calcium dynamics. Literature indicates that calcium signaling is a critical indicator of neuronal functionality, with increased calcium activity reflecting successful neuronal differentiation and maturation [42, 54]. The strong correlation observed between GNP concentration and enhanced calcium fluorescence intensity supports the notion that GNPs facilitate the functional maturation of neurons by promoting calcium influx and excitability. The observed initial enhancement of calcium signaling in response to ATP stimulation, followed by a reduction in calcium density by day 7 in GNP-coated conditions, suggests a complex regulation of calcium homeostasis during neuronal differentiation. This pattern aligns with the understanding that calcium signaling plays a pivotal role in neuronal maturation and functional stability. Disruptions in calcium homeostasis can adversely affect neurite branching and arborization, which are critical phases in neuronal development [55]. Additionally, ATP-induced calcium-signaling mechanisms are integral in mediating various aspects of neuronal differentiation and function [56]. Therefore, the modulation of calcium dynamics observed in our study reflects the intricate balance required for proper neuronal development and the establishment of functional neural networks.

In conclusion, our study systematically investigated the influence of GNP coatings on hBMSC viability, neuronal differentiation, and functional maturation, integrating molecular, morphological, and functional assessments. We successfully generated a stable GFP-expressing hBMSC line to track neuronal differentiation. Among all tested conditions, 0.4 µg/ml GNP (with 0.2% gelatin) was identified as the most effective concentration for promoting neuronal differentiation, extensive network formation, and functional maturation. However, 0.2 µg/ml GNP also demonstrated promising effects with potentially lower cytotoxicity for applications requiring prolonged cell viability. Functional assessment via Ca^2+^ imaging revealed that neurons cultured on 0.2 and 0.4 µg/ml GNP exhibited enhanced ATP-induced calcium transients at day 4, followed by a reduction at day 7, suggesting a critical transition toward functional homeostasis and network stabilization. This adaptive response indicates that GNPs not only accelerate neuronal differentiation but also contribute to calcium homeostasis, a key factor in preventing excessive excitability and ensuring long-term neuronal stability. This study represents a pioneering and novel approach in neural tissue engineering by demonstrating that graphene nanoplatelets can serve as a biofunctional platform to induce neuronal differentiation from hBMSCs. Given their conductive, biocompatible, and differentiation-enhancing properties, GNP-based coatings could be further explored for biomedical applications such as neural regeneration, bioelectronic interfaces, and stem cell-based therapies. Future research should focus on electrophysiological validation, in vivo integration, and optimizing GNP formulations for clinical translation, advancing the potential of graphene-based biomaterials in neuroregenerative medicine.

## Supplementary Information

Additional file 1.

## Data Availability

All data generated or analyzed during this study are included in this published article (and its supplementary information files) or are available from the corresponding author upon request.
