# Supplementary material for: Graphene nanoplatelets enhance neuronal differentiation of human bone marrow mesenchymal stem cells
Source: Biol Res. 2025 May 30;58:32. doi: 10.1186/s40659-025-00616-3 (PMC12123866; doi:10.1186/s40659-025-00616-3)
Supplement: Supplementary file 1 — Additional file 1. [file 40659_2025_616_MOESM1_ESM.docx]

**SUPPLEMENTARY FIGURES**

**
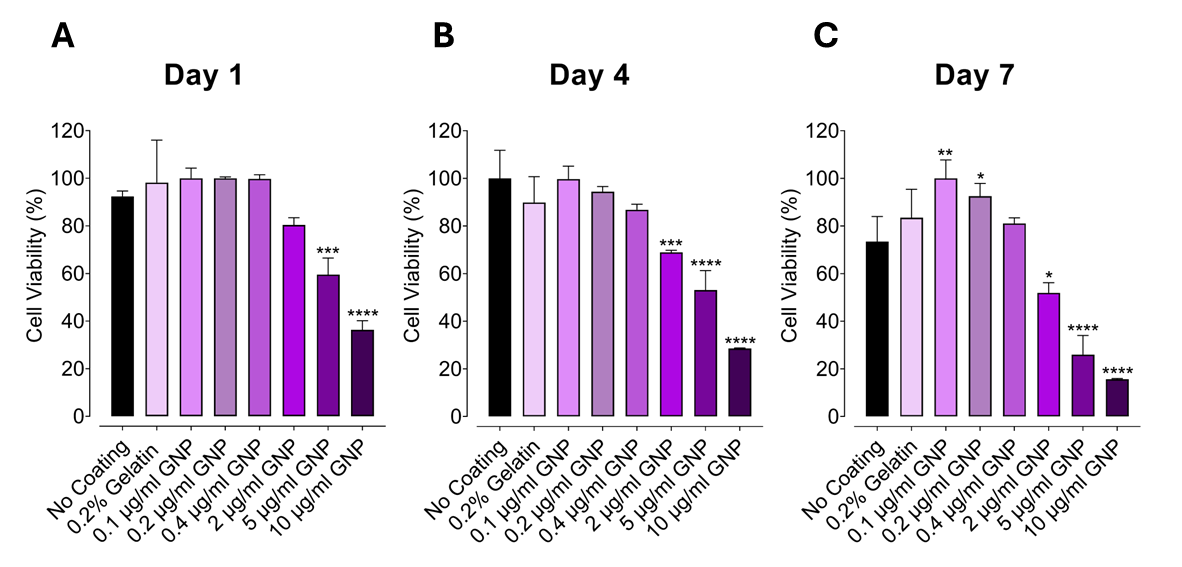
**

**Supp. Figure 1. Effect of GNP concentrations on hBMSCs viability over time.** Bars represent MTT assay results on **(A)** day 1, **(B)** day 4, and **(C)** day 7. Black bars denote control (no coating), light purple bars represent 0.2% gelatin coating, and other bars correspond to increasing GNP concentrations (0.1 to 10 µg/ml) with 0.2% gelatin. Data are presented as mean ±SD (n=3). Statistical significance was determined using Dunnett’s test relative to the control column. (A) ****p=0.0003*, *****p<0.0001;* (B) ****p=0.0002*, *****p<0.0001*; (C) **p=0.0295*, **p=0.0130*, ***p=0.0024*, *****p<0.0001*.

**
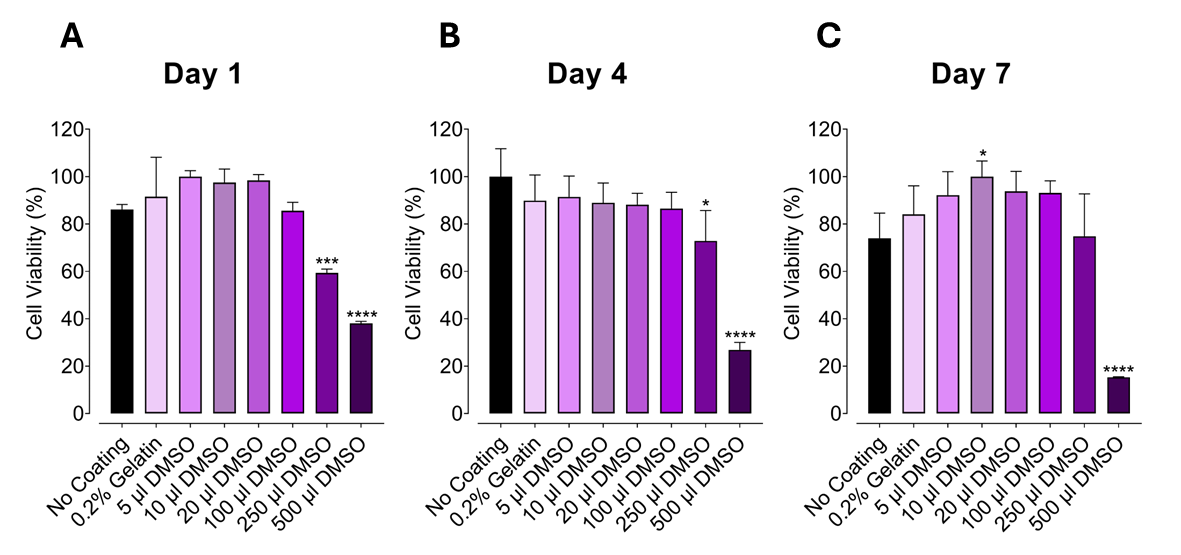
**

**Supp. Figure 2. Effect of DMSO concentrations (from GNP solutions) on hBMSC viability over time.** Bars represent MTT assay results on **(A)** day 1, **(B)** day 4, and **(C)** day 7 without GNP addition. Black and light purple bars denote conditions without DMSO addition, while other bars indicate increasing DMSO concentrations (without GNP), all with 0.2% gelatin. Data are presented as mean ±SD (n=3). Statistical significance was determined using Dunnett’s Test relative to the control column (black bar): (A) ****p= 0.0007*, *****p< 0.0001;* (B) **p=0.0108, ****p<0.0001*; (C) **p=0.0321, ****p<0.0001*.


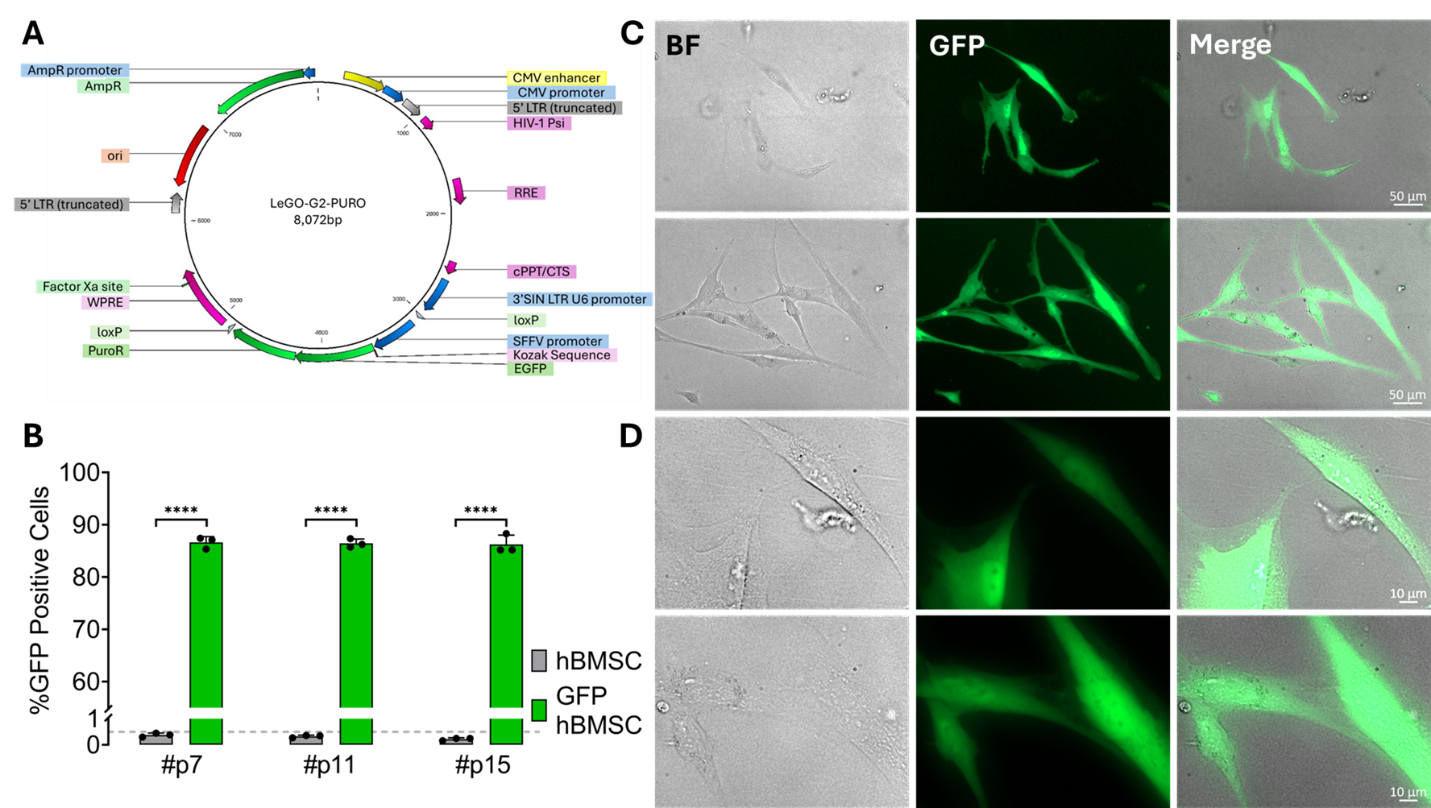


**Supp. Figure 3. Lentiviral transduction of hBMSCs with the LeGO-G2-PURO vector for stable GFP expression. (A)** Plasmid map of LeGO-G2-PURO lentiviral vector (8,072 bp) contains an SFFV promoter-driven EGFP gene for stable GFP expression. **(B)** Flow cytometry analysis of GFP-positive hBMSCs post-transduction. Data are presented as mean ±S.D. Statistical significance was determined using Bonferroni’s test, *****p<0.0001*. **(C)** Wide-field images (20x objective) show GFP expression and overall cell morphology. **(D)** High-magnification images (63x objective) provide detailed visualization of intracellular GFP distribution.


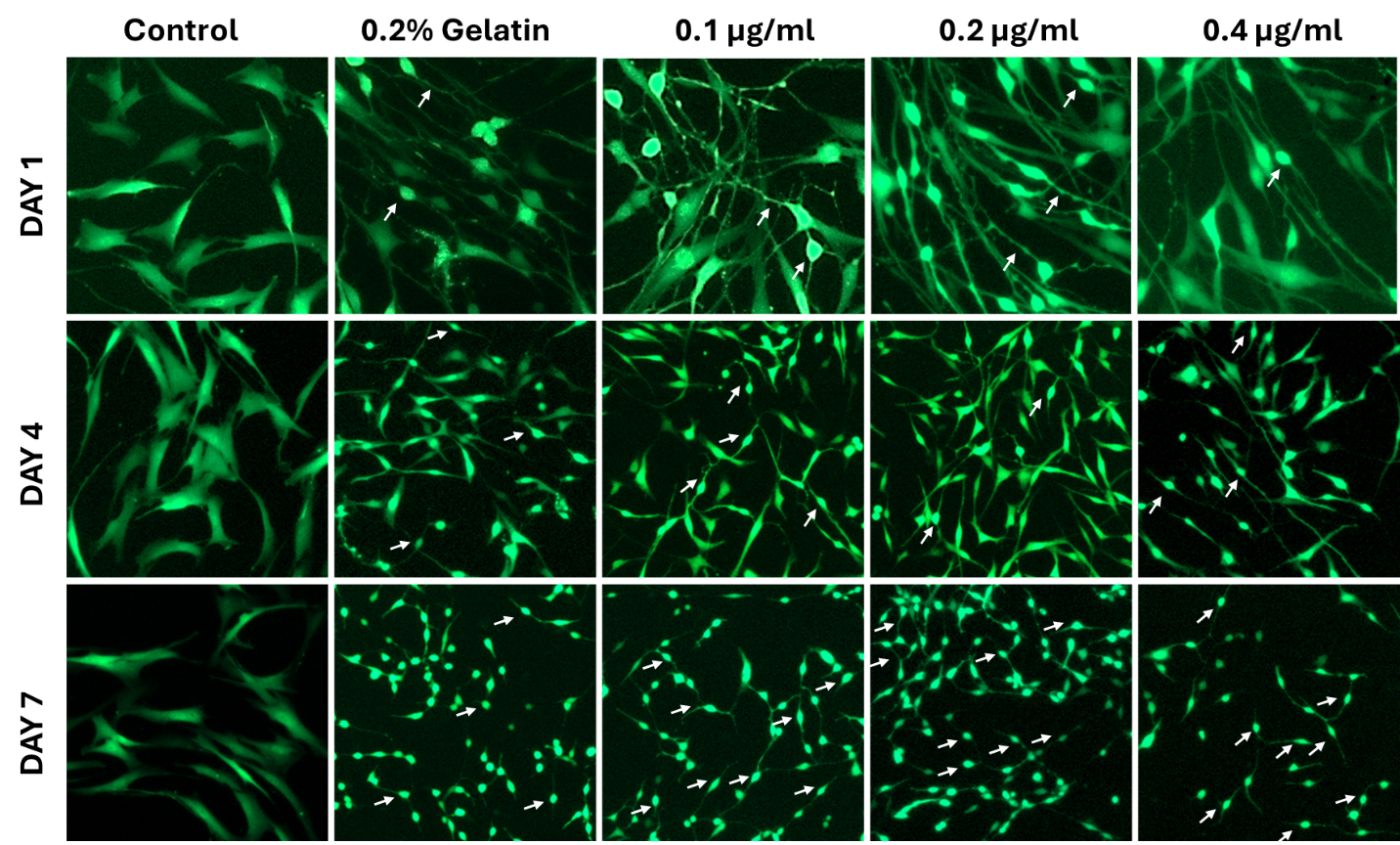


**Supp. Figure 4. Digitally zoomed fluorescence images of GFP-expressing hBMSCs during neuronal differentiation.** These images correspond to Figure 1B, providing a closer view of hBMSCs cultured under different conditions over time. The zoomed-in images highlight morphological changes, including neuron-like elongation. White arrows highlight the gradual formation of neurite-like extensions and interconnected networks.
